# Exercise, Diet and Sleeping as Regenerative Medicine Adjuvants: Obesity and Ageing as Illustrations

**DOI:** 10.3390/medicines9010007

**Published:** 2022-01-14

**Authors:** Abdelaziz Ghanemi, Mayumi Yoshioka, Jonny St-Amand

**Affiliations:** 1 Functional Genomics Laboratory, Endocrinology and Nephrology Axis, CHU de Québec-Université Laval Research Center, Québec, QC G1V 4G2, Canada; abdelaziz.ghanemi@crchudequebec.ulaval.ca (A.G.); mayumi.yoshioka@crchudequebec.ulaval.ca (M.Y.); 2 Department of Molecular Medicine, Faculty of Medicine, Laval University, Québec, QC G1V 0A6, Canada

**Keywords:** regeneration, exercise, diet, sleeping, oxidative stress, inflammation, obesity, ageing

## Abstract

Regenerative medicine uses the biological and medical knowledge on how the cells and tissue regenerate and evolve in order to develop novel therapies. Health conditions such as ageing, obesity and cancer lead to an impaired regeneration ability. Exercise, diet choices and sleeping pattern have significant impacts on regeneration biology via diverse pathways including reducing the inflammatory and oxidative components. Thus, exercise, diet and sleeping management can be optimized towards therapeutic applications in regenerative medicine. It could allow to prevent degeneration, optimize the biological regeneration and also provide adjuvants for regenerative medicine.

## 1. Regeneration and Medicine

Regeneration can be defined as the biological processes allowing the cells, organs and tissues to renew and proliferate. These processes also allow normal growth and development, maintenance of a healthy body [1] as well as the recovery from injuries [2] or from other external perturbations [3]. It involves various underlying pathways such as cytoprotective mechanisms induction [2], cellular plasticity [4], tissue remodeling [5] and biophysical aspects [6]. Impaired regeneration can have pathological impacts such as degenerative diseases in different tissues [7,8,9,10]. These diseases result from the loss of the regenerative ability leading to a status where cellular loss is superior to cellular regeneration. Regeneration processes might also be impaired or disturbed in various status including degenerative diseases [11,12,13], cancer [14], obesity [15], ageing [16,17], diabetes mellitus [18] and cholestatic liver [19]. Regenerative medicine comes as a branch that aims to understand the regenerative pathways and degenerative processes, both in biology and physiology, to develop methodologies and approaches aiming to correct regeneration-related health challenges and the impaired functions [20] or at least limit the impacts of the variables that can impair regeneration. The regenerative medicine is a medical field based on regeneration, used biomaterials [21], biochemistry [22], stem cells [23,24] and tissue engineering [25,26] and applies them in surgery [27], transplantation [28], ophthalmology [29] and cancer [23] among diverse applications [30,31,32,33,34,35].

Regenerative medicine, based on regenerative biology [36], aims to elucidate the mechanistic pathways underlying cellular and tissular regeneration along with the endogenous and exogenous factors that influence the regenerative processes and use that knowledge to develop novel therapeutic options. Such therapies target the correction or the optimization of an impaired regeneration resulting from a disease, a physiological adaptation or even therapeutic side effects. Regenerative medicine research involve multiple areas from stem cells [31], gene editing [37], nuclear transfer [38], proteomics, pharmacology, nanotechnology [39], tissue, engineering and three-dimensional printing [40]. Beside the various adjuvant used in regenerative medicine, mainly pharmacological (regenerative pharmacology) [41,42,43] or bioengineering [28,44], we aim to highlight the importance of lifestyle and how it impacts regeneration. In this piece of writing, we would like to provide illustrative examples on how lifestyle patterns—specifically exercise, diet and sleeping—influence regeneration and the related biological processes. We also present potential clinical and biomedical applications.

## 2. Exercise, Diet, Sleeping and Regeneration

The three main lifestyle pillars (exercise, diet and sleeping) represent key factors in regeneration and, thus, in regenerative medicine as we illustrate below. The facts that they impact regeneration and also influence the statuses (obesity, ageing, etc.) in which regeneration patterns change, support that exercise, diet and sleeping as key factors worth exploring to optimize regeneration medicine applications. In addition, exercise, diet and sleeping are also involved in different physiological changes and pathological prognoses.

Exercise, is known for numerous health benefits including metabolic enhancement [45,46,47] and regenerative induction. Indeed, exercise represents a cardiomyocyte regeneration inducer [48,49], a therapeutic cartilage regeneration adjuvant [50], a skeletal muscle regeneration enhancer [51], and a cardiac remodeling inducer [52]. Exercise might/can also slow down [53] or reverse muscle atrophy [54], improve the post-injury skeletal muscle regeneration [55], prevent stem cells senescence [56], promote peripheral nerve regeneration [57], and rejuvenate muscle stem cells [58,59]. Within the context of the mechanisms underlying the exercise-induced regenerative benefits, secreted protein acidic and rich in cysteine (SPARC) is at the center of a key theory. SPARC is both induced by exercise and has been hypothesized as a regeneration factor [60,61,62]. Such implication in regeneration enhancement would be explained by the various properties and roles it has [63] including anti-inflammatory [64], antitumor [61,65,66], extracellular matrix structure [67] and metabolism [68,69] in addition to studies linking SPARC to regeneration [70,71] as well as potential applications in personalized medicine [72]. This suggested that at least a part of the benefits induced by exercise are mediated by SPARC. Indeed, we have already presented data suggesting that that exercise-induced muscle phenotype changes are SPARC-dependent [73] which is in accordance with the theory linking myokines to the physical activity effects [66,74]. The positive impacts of exercise on regeneration could be explained by the properties that have been associated to exercise, such as anti-inflammatory [75], antioxidant [76], anticancer [77] and anti-ageing [78] properties, that lead to suitable outcomes for regeneration and bio-homeostasis in general.

Diet, an important determinant of health, has been studied in a variety of contexts including obesity, metabolism and cancer [79]. However, and although diet and nutrition have been exploited for tissue regeneration [80], many details of the molecular mechanistic pathways seem still emerging to light. The choice of diet quality as well as fasting (calorie restriction) [81] do impact regeneration. Diverse examples illustrate how dietary choices could impact regeneration. Supplemented nutrition diet affects regeneration in liver [82], high-fat and high-glucose microenvironment inhibits bone regeneration [83], proliferation and migration of human gingival fibroblasts is impaired by high glucose-induced oxidative stress [84] but following lidocaine-induced injury normal glucose enhances neuronal regeneration [85]. Such links between glucose and regeneration could be behind parts of the regeneration patterns seen during diabetes [86].

Another illustration in the same context is that fasting promotes stem cell-based regeneration [87], promotes intestinal regeneration [88,89], promote hematopoietic-stem-cell-based regeneration [90] and β-cell regeneration [91,92]. Such fasting benefits on regeneration would involve metabolic and body composition changes [87,93], anti-inflammatory effects [89], stem cell number increase [88], oxidative stress decrease and ageing delaying [94,95]. The dietary choices represent an important player as well. The rationale behind the dietary choice is to generate a biological microenvironment that can promote regeneration. This could be achieved, for instance, by reducing the intake of the high-fat diet since high-fat diet leads to inflammation [96,97] and cancer progress [96,98]. The other way to improve regeneration environment via diet is to create biological conditions that would optimize the regenerative abilities. This can be achieved with diets that provide properties such as antioxidant [99,100], anti-inflammatory [101,102], omega-3 fatty acids [103], protein intake [104] and microbiota composition change [105,106]. For instance, fasting-mimicking diet promotes intestinal regeneration [88], reduces intestinal inflammation [89] and reduce inflammatory bowel disease pathology [88].

The other pillar of lifestyle is represented by the sleeping wish is neuroprotective [107]. Impact of sleep on stem cell regenerative capacity is shown by the correlation between circadian rhythm and an improved stem cells proliferation microenvironment [108] leading to stem cell maintenance and division control [109,110]. This fits with the melatonin anti-inflammatory, antioxidant and neuroprotective properties [111,112] along with its free radical scavenger function [113], among others, that would be behind its role in regeneration of tissues [111,113]. In addition, protein and pre-sleep are also contributors to regeneration [114,115]. Following the same line of thoughts, sleep deprivation impairs muscle regeneration [116] and delays healing process [117] which supports the importance of sleeping for the regenerative processes.

These examples of the implications of exercise, diet and sleeping at various levels in regeneration and its variables clearly show their importance within any intervention aiming to stimulate or modulate regeneration.

## 3. Obesity and Ageing as Selected Illustrations

Beside the known diseases related to regeneration changes, obesity [118] and ageing [119] represent topics of concern and are health conditions worth exploring in the context of regeneration. They both share common biological and pathological features [120,121] including regeneration-related [122]. The focus on obesity and ageing, that have common patterns [120,123], derives from their globally increasing epidemiological profile along with the diseases and health problems related to them, including those impacting regeneration homeostasis.

Obesity, as a neuroendocrine reprogramming [124], represents a status of a broken energy balance [125] that has been classified as a disease [126,127,128]. It has been associated with various health problems and diseases [129]. In the ongoing COVID-19 pandemic, it is worth pointing that obesity both increases vulnerability to COVID-19 (vicious cycle [130]) and reduces the immunity [131,132]. Ageing, on the other hand, can be defined as the time-dependent biological and functional declines of living entities. It represents a risk factor for various diseases too [133,134]. Ageing has a genetic component [135] and is due—at least in part—to hormonal and metabolic changes [136]. At the molecular levels, epigenetic changes such as DNA methylation [137,138] are involved in age-related changes. Whereas obesity is a status in which regeneration is impaired [15,139], ageing is also accompanied by a decline in regeneration [59,140,141,142]. Studies and hypotheses have pointed various age-related underlying mechanism such as the loss of biological plasticity [143] and the changes in the regenerative environment [141].

The prescription of exercise for both obese [144,145] and elderly population [146,147,148] is well documented. Such prescription is based on the numerous benefits exercise has; among which we cite glycemic control [149,150], weight management [145], antioxidant [76], anti-inflammatory [151,152], cardiovascular risks improvement [153], immune system regulation [154] and anti-inflammatory milieu promotion [49]. All these benefits improve the negative consequences induced by ageing and obesity and, importantly, improve regeneration bioenvironment.

Exercise is prescribed in obesity and ageing for reasons initially independent from regeneration (weight and adiposity loss, muscle function improvement, etc.). However, the above examples clearly reflect how exercise would be important for regeneration, including in the contexts of obesity and ageing. Exercise would both improve regeneration and reduce the negative impacts that obesity and ageing have on regeneration. The dietary choices and sleeping patterns described above would also contribute to reduce the impacts of obesity and ageing as well. Therefore, indirectly improve the biological regeneration ability. Importantly, targeting regeneration-related pathways in both obesity and ageing represents an additional shared pattern between obesity and ageing.

## 4. Perspectives

**Figure 1:**
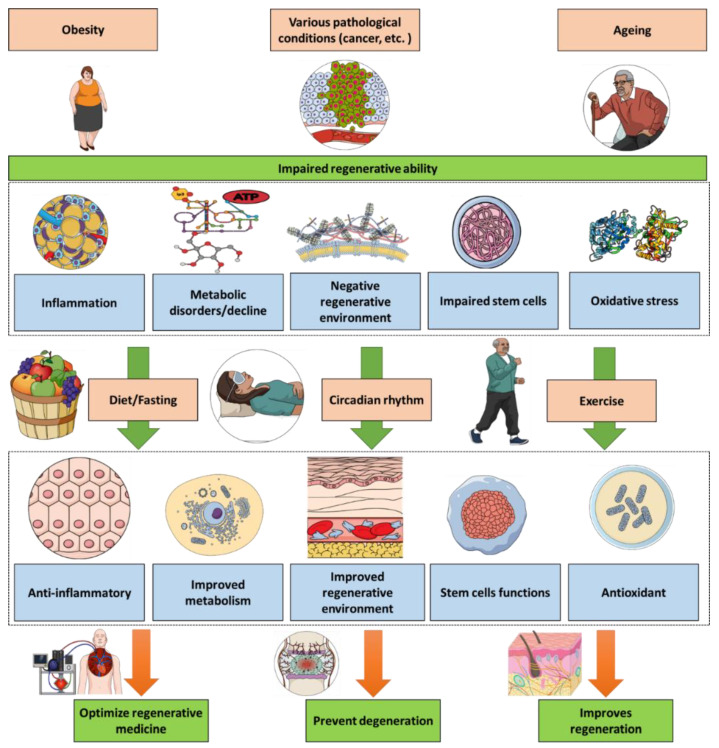
Health conditions such as ageing, obesity and cancer lead to an impaired regenerative ability. Exercise, diet and sleeping have significant impacts on regeneration biology via diverse pathways including reducing the inflammatory and oxidative components. Thus, exercise, diet and sleeping management can be optimized towards therapeutic applications in regenerative medicine.

The above illustrative examples point to the importance of exercise, diet and sleeping within the regenerative context and points the important of combining all these factors to reach the optimum regenerative outcome. This would have two key implications (Figure 1). First, developing an unhealthy lifestyle could lead to both regenerative problems and a possible therapeutic failure of the regenerative medical therapies. The second implication is the importance of introducing medically supervised choices for exercise, diet and sleeping patterns as a regenerative adjuvants either during regenerative therapies or for individuals suffering from conditions impacting the regenerative abilities such as obese and elderly patients, knowing the shared features between ageing and obesity [123].

However, further studies are required in order to identify the quality and the quantity of each of these three elements and their combination for each specific case. Indeed, the choice of exercise patterns (types of exercise, duration, timing [150], etc.), diet (quantity, composition and timing) as well as sleeping (duration, timing and tissue-specific impacts [155]) are parameters for which additional optimization could improve the use and application of exercise, diet and sleeping as therapeutic adjuvants or even as stimulators for regeneration. This is encouraging considering the recent advances in biology, such as the possible regenerative ability of the adult heart [156]. Molecular tools such as functional genomics [157,158,159,160,161,162,163] and metabolics would allow the characterization of diverse genes, proteins and other molecular and biochemical changes related to exercise, diet and sleeping patterns, along with their implications in regeneration as well understanding regeneration via proteomics [164]. This would elucidate the molecular links and, thus, identify potential novel pharmacological targets based on advances in signaling in regeneration [165]. These targets are of a specific importance since they would allow, for instance, to mimic exercise effects via pharmacological agents without the need of performing exercise. Such an approach is important for individuals who have a limited ability to complete the prescribed physical activities, such as elderly patients and those with physical disabilities.

Overall, the effects of a healthy lifestyle (exercise, diet and sleeping) all contribute towards an improved regeneration ability, which is required to improve healthy ageing, especially with regard to the increased human lifespan [166], in addition to all the known benefits of a healthy lifestyle for a limitless number of health problems including diabetes [167], cancer [168], mental health [169], pediatric asthma [170] and reproductive health [171]. Although we have focused on the impacts exercise, diet and sleeping would have on the biology of regeneration in vivo, we can also extrapolate the concept towards possible in vitro applications. Within this context, the cytokines and factors induced by exercise (ex. SPARC or use the in vitro model of exercise [172]) and sleeping (ex. melatonin) as well as the diet chemical components (ex. antioxidant) could be supplemented during the bioengineering of cellular and tissular cultures (adding to the cells and tissues medium) to enhance the growth and optimize the regenerative abilities (ex. stem cells therapy) prior of their introduction in the organism as a regenerative medical therapy.
